# Directional Topography Influences Adipose Mesenchymal Stromal Cell Plasticity: Prospects for Tissue Engineering and Fibrosis

**DOI:** 10.1155/2019/5387850

**Published:** 2019-05-05

**Authors:** Gabriel Romero Liguori, Qihui Zhou, Tácia Tavares Aquinas Liguori, Guilherme Garcia Barros, Philipp Till Kühn, Luiz Felipe Pinho Moreira, Patrick van Rijn, Martin C. Harmsen

**Affiliations:** ^1^University of Groningen, University Medical Center Groningen, Department of Pathology and Medical Biology, Groningen, Netherlands; ^2^Laboratório de Cirurgia Cardiovascular e Fisiopatologia da Circulação (LIM-11), Instituto do Coração (InCor), Hospital das Clinicas HCFMUSP, Faculdade de Medicina, Universidade de Sao Paulo, Sao Paulo, SP, Brazil; ^3^University of Groningen, University Medical Center Groningen, Department of Biomedical Engineering-FB40, W.J. Kolff Institute for Biomedical Engineering and Materials Science-FB41, A. Deusinglaan 1, 9713 AV, Groningen, Netherlands; ^4^Institute for Translational Medicine, State Key Laboratory of Bio-fibers and Eco-textiles, Qingdao University, Qingdao 266021, China

## Abstract

**Introduction:**

Progenitor cells cultured on biomaterials with optimal physical-topographical properties respond with alignment and differentiation. Stromal cells from connective tissue can adversely differentiate to profibrotic myofibroblasts or favorably to smooth muscle cells (SMC). We hypothesized that myogenic differentiation of adipose tissue-derived stromal cells (ASC) depends on gradient directional topographic features.

**Methods:**

Polydimethylsiloxane (PDMS) samples with nanometer and micrometer directional topography gradients (wavelength (*w*) = 464-10, 990 nm; amplitude (*a*) = 49-3, 425 nm) were fabricated. ASC were cultured on patterned PDMS and stimulated with TGF-*β*1 to induce myogenic differentiation. Cellular alignment and adhesion were assessed by immunofluorescence microscopy after 24 h. After seven days, myogenic differentiation was examined by immunofluorescence microscopy, gene expression, and immunoblotting.

**Results:**

Cell alignment occurred on topographies larger than *w* = 1758 nm/*a* = 630 nm. The number and total area of focal adhesions per cell were reduced on topographies from *w* = 562 nm/*a* = 96 nm to *w* = 3919 nm/*a* = 1430 nm. Focal adhesion alignment was increased on topographies larger than *w* = 731 nm/*a* = 146 nm. Less myogenic differentiation of ASC occurred on topographies smaller than *w* = 784 nm/*a* = 209 nm.

**Conclusion:**

ASC adherence, alignment, and differentiation are directed by topographical cues. Our evidence highlights a minimal topographic environment required to facilitate the development of aligned and differentiated cell layers from ASC. These data suggest that nanotopography may be a novel tool for inhibiting fibrosis.

## 1. Introduction

The generation of tissue-engineered blood vessels (TEBV) is facilitated by the use of mesenchymal stem cells (MSC) as precursors for vascular smooth muscle cells (SMC), i.e., the media layer of TEBV [1–5]. These highly plastic MSC, originating from the mesodermal embryonic tissue and present in all connective tissues of the adult human body, are relatively easy to isolate, cultivate, and characterize [6, 7]. Of note, because all MSC are derived from the stromal (vascular) fraction of tissue, the consensus is to name these mesenchymal stromal cells. Stromal cells have the propensity to differentiate but lack self-renewal, in contrast to genuine stem cells. One particular type of MSC is the adipose tissue-derived stromal cell (ASC). In addition to the characteristics common to MSC, ASC have a number of advantages as a source of precursor cells for the production of TEBV: they are easy to acquire, culture, propagate, and differentiate [8–12].

MSC share characteristics with fibroblasts presenting similar cell behaviors [13–16]. Activated fibroblasts in turn are involved in the fibrotic process through myofibroblast differentiation. This process is primarily driven by transforming growth factor beta (TGF-*β*) and contributes to the profibrotic microenvironment [17–22]. During TGF-*β*-induced MSC differentiation to SMC, MSC pass through the transitory state of being a “myofibroblast”—expressing smooth muscle protein 22 alpha (SM22*α*) and alpha smooth muscle actin (*α*SMA)—before maturing to SMC that express smooth muscle myosin heavy chain (SM-MHC) protein. During this intermediate immature stage, MSC behave as myofibroblasts.

In order for complete MSC differentiation into mature SMC, mechanical stimulation is a recognized key facilitatory factor [23, 24]. It has been shown that uniaxial strain activates mechanosensitive pathways which regulate cellular behaviors thus inducing increased myogenic markers (SM22*α*, *α*SMA, and SM-MHC) [25–28]. Equiaxial strain, on the other hand, showed the opposite effect, with downregulation of these markers [27]. The optimal setup for MSC-derived SMC maturation requires the use of perfusion bioreactors in 3D tubular constructs. By adopting this approach, previous studies from our group and others have demonstrated that MSC not only spontaneously differentiate into SMC, i.e., without the need for TGF-*β* [12], but also deposit the elastic components of vascular extracellular matrix [29]. The construction and handling of such bioreactors, however, can be laborious, expensive, and time-consuming. To overcome this limitation, the harnessing of the regulation of the physical topographical properties of biomaterials to direct cell fate might be a promising alternative.

It is recognized that the culture of stem and progenitor cells on biomaterials with specific physical topographical properties influences their alignment and differentiation [30–34]. The literature on the influence of surface topography on myogenic differentiation is limited, e.g., adipogenic differentiation of MSC is promoted by larger directional topographies [35–37], while osteogenic differentiation is promoted by smaller features [35, 36], when these were compared in different feature ranges. So far, the assessment of MSC adhesion, alignment, and myogenic differentiation has been restricted to a few topographies [37–41]. Therefore, the differences in cell behaviors if MSC are cultured on variably aligned topographic dimensions are partly elucidated. Strengthening this understanding could be a key tool for optimizing the harnessing of biomaterials for, on the one hand, inhibition of fibrotic processes and on the other hand vascular tissue engineering.

In our study, we aimed to investigate the influence of gradient wrinkle features on the adhesion, alignment, and myogenic differentiation of ASC. Unidirectional gradients were studied with variable wavelength and amplitude from nano- to microscale. In addition, we assessed the influence of stimulation with TGF-*β*1 on myogenic differentiation.

## 2. Experimental Section

### 2.1. Fabrication and Characterization of Topography Substrates for Cell Culture

Polydimethylsiloxane (PDMS) elastomer samples were obtained by mixing the prepolymer and the crosslinker (SYLGARD 184 kit, Dow Corning, MI, USA) at a 10 : 1 ratio by mass according to the supplier's information. The mixture was vigorously stirred with a spatula, and vacuum was applied for 15 min to degas the mixture. Subsequently, the mixture was deposited on a carefully cleaned planar polystyrene petri dish at a thickness of ~1.5 mm, before curing at 70°C in a vacuum oven overnight to crosslink into an elastomer.

For high-throughput screening purposes, nano- and microlinear gradients were fabricated. For further investigation of the gene and protein expressions, two uniform directional topography setups were fabricated, according to the results of the screening phase of the study. The fabrication process, which was based on previously published methods with minor modifications [42, 43], is described below.

#### 2.1.1. Generating Gradient Linear Topographies

**Figure 1:**
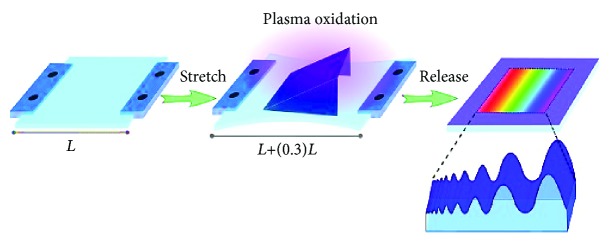
Schematic illustration of the operational process for the fabrication of gradient linear topographies using PDMS via prolonged plasma oxidation. Adapted from “Screening Platform for Cell Contact Guidance Based on Inorganic Biomaterial Micro/nanotopographical Gradients” by Zhou et al. [57]. Adapted with permission.

Gradient linear topographies were fabricated for immunofluorescence screening. PDMS samples were cut into strips of 3.0 × 1.5 cm. The strips were stretched uniaxially in a custom-made apparatus to a strain of 130% of their original length. For gradient directional topography formation, stretched PDMS samples were partly covered with a one-side opened mask (for nanotopography gradient: angle = 45°, length = 1.3 cm; for microtopography gradient: angle = 30°; length = 1.3 cm) and oxidized (for nanotopography gradient: 100 s at 60 mTorr; for microtopography gradient: 650 s at 25 mTorr) at maximum intensity (Atto Plasma System, Diener Electronic GmbH, Ebhausen, Germany). Upon removing the stress, gradient directional topography formation with different wavelengths and amplitudes was formed.  Figure 1 illustrates the operational process for the fabrication of gradient linear topographies. All samples were posttreated with plasma for 10 min at 100 mTorr and maximum intensity to ensure that the surfaces were fully oxidized and that surface chemistry, as well as stiffness, was equal. Treated PDMS strips were cut in circles of 14 mm in diameter, immersed in 70% ethanol for sterilization, and placed in 24-well tissue culture plates for cell culture. For all experiments, fully oxidized planar PDMS samples were used as controls.

#### 2.1.2. Uniform Linear Topographies

Uniform linear topographies were fabricated for analyzing cellular responses by RT-qPCR and WB analysis. PDMS samples were cut into a 9.0 × 9.0 cm square sample and stretched uniaxially in a custom-made apparatus to a strain of 130% of their original length. Stretched PDMS samples were oxidized (for 1 *μ*m uniform features: 250 s at 14 Torr; for 11 *μ*m uniform features: 650 s at 20 mTorr) at maximum intensity (Plasma Activate Flecto 10 USB). At completion, the stress was removed, inducing wrinkle formation with uniform features. All samples were posttreated with plasma for 10 min to ensure that the surfaces were fully oxidized and that surface chemistry, as well as stiffness, was equal. Treated PDMS strips were cut in circles of 22 mm in diameter, immersed in 70% ethanol for sterilization, and placed in 12-well tissue culture plates for cell culture. For all experiments, fully oxidized planar PDMS samples were used as controls.

#### 2.1.3. Characterization of Topography Substrates

Surface topography of patterned PDMS samples was analyzed by atomic force microscopy (AFM) using a commercial atomic force microscope (NanoScope V Dimension 3100 microscope, Veeco, USA) operating with contact mode in air. The wavelength and amplitude of directional topographies in the captured images were analyzed by NanoScope Analysis software. The chemical composition of the PDMS surfaces was determined using X-ray photoelectron spectroscopy (XPS) (S-Probe, Surface Science Instruments, Mountain View, USA) equipped with an aluminum anode. Samples were placed in the prevacuum chamber of the XPS and then subjected to a vacuum of 10^−9^ Pa. X-rays (10 kV, 22 mA), at a spot size of 250 × 1000 *μ*m, were produced using the aluminum anode. Scans of the overall spectrum in the binding energy range of 1-1100 eV were made at low resolution (pass energy 150 eV).

### 2.2. Cell Sources and Culture

#### 2.2.1. Cell Sources

Adipose tissue-derived stromal cells (ASC) were isolated from human subcutaneous fat acquired through liposuction (Bergman Clinics, the Netherlands). The use of adipose tissue as a source of ASC was approved by the local Ethics Committee of the University Medical Centre Groningen, given that the material was considered as anonymized waste material. For all the anonymous donations, patients had provided written informed consent as part of their surgical admission procedure.

ASC were isolated as previously described [12, 44, 45]. Briefly, collected adipose tissue was extensively washed with phosphate-buffered saline (PBS) buffer to remove blood; then tissue was enzymatically digested with 0.1% collagenase A (Roche Diagnostic, Mannheim, Germany) in PBS containing 1% bovine serum albumin (BSA) (Sigma-Aldrich, St. Louis, United States), at 1 : 1 ratio of tissue to digestion solution, with shaking at 37°C for 1 h. Digested tissue was filtered and washed with 1% BSA/PBS solution. Collected cells were suspended and cultured in Dulbecco's modified Eagle's medium (DMEM) (Lonza BioWhittaker, Verviers, Belgium) supplemented with 10% fetal bovine serum (FBS) (Thermo Scientific, Hemel Hempstead, UK), 1% L-glutamine (Lonza BioWhittaker, Verviers, Belgium), and 1% penicillin/streptomycin (Gibco Invitrogen, Carlsbad, United States) at 37°C in a humidified incubator with 5% CO_2_. The medium was refreshed three times per week until cells had reached sufficient confluency.

#### 2.2.2. Culture Protocol

Cells between passages 3 and 5 were seeded in culture plates at 5000 cells/cm^2^ and 10000 cells/cm^2^ seeding densities, respectively, for cell behavior screening and further comparisons between the uniform topographies. Cells were cultured in Dulbecco's modified Eagle's medium (DMEM) (Lonza) supplemented with 10% fetal bovine serum (FBS) (Thermo Scientific, Hemel Hempstead, United Kingdom), 1% L-glutamine (Lonza BioWhittaker, Verviers, Belgium), and 1% penicillin/streptomycin (Gibco Invitrogen, Carlsbad, United States) at 37°C in a humidified incubator with 5% CO_2_. In a series of experiments, ASC were treated with 1 ng/ml TGF-*β*1 (PeproTech, London, United Kingdom) in culture medium for seven days, as previously described [12].

### 2.3. Immunofluorescence, Gene Expression, and Immunoblotting

#### 2.3.1. Immunofluorescence

Adhesion and alignment of cells were assessed after 24 h. Cells were fixed with 2% paraformaldehyde in PBS for 30 min, washed with PBS twice, permeabilized with 0.5% Triton X-100 in PBS for 15 min, and blocked with 1% BSA and 5% donkey serum solution in PBS for 15 min to avoid nonspecific binding. Samples were incubated with mouse anti-vinculin primary antibody (1 : 100; Sigma-Aldrich, St. Louis, United States) for 2 h, washed with 0.05% Tween in PBS, and incubated again with goat-anti-mouse IgG FITC-labelled secondary antibody (1 : 100; green; Jackson ImmunoLabs, West Grove, United States) for 1 h at room temperature. For F-actin, cells were stained with Phalloidin-TRITC (2 *μ*g/ml; red; Sigma-Aldrich, St. Louis, United States) and nuclei were stained with 4′,6-diamidino-2-phenylindole (DAPI) (4 *μ*g/ml; blue; Sigma-Aldrich, St. Louis, United States). Samples were washed three times with PBS and stored in the dark for image acquisition. Fluorescently stained samples were imaged using a TCS-SP2 Confocal Laser-Scanning Microscope (Leica, Wetzlar, Germany), 40x magnification NA 0.80 water immersion objective, and for cell alignment using the TissueFAXS microscopy system (TissueGnostics, Vienna, Austria), 10x magnification. All images were collected with the same hardware and software settings of the microscopy system; exposure time and gain were kept constant for each experiment. Overviews of all the samples were obtained. Each of the two topography patterns were divided into five (nano) or six (micro) ranges (R1-R5 for nanotopography and R6-R11 for microtopography) through division of each of the two image overviews into five or six micrographs for analysis, totaling 11 pictures representing the 11 ranges.

Micrographs of immunofluorescence staining for vinculin were analyzed using the Focal Adhesion Analysis Server [46] and ImageJ software to measure the number of focal adhesions per cell, the area of each single focal adhesion, the total focal adhesion area per cell, and the focal adhesion alignment to the directional topography. Alignment analysis was done using ImageJ software, and cells were considered aligned if the angle between their long axis and the wrinkle was less than 10° (the smaller the angle, the higher the alignment), according to previously established references [47].

Myogenic differentiation of ASC was assessed after 7 days. Samples were incubated with rabbit anti-SM22*α* primary antibody (1 : 800; Abcam, Cambridge, United Kingdom) for 2 h, washed with 0.05% Tween in PBS, and incubated again with donkey anti-rabbit Alexa Fluor® 594 secondary antibody (1 : 800; red; Abcam, Cambridge, United Kingdom) for 1 h at room temperature. Cytoskeleton was stained by Phalloidin Alexa Fluor® 488 (1 : 400; green; Thermo Fisher, Waltham, United States), and nuclei were stained with DAPI (4 *μ*g/ml; blue; Sigma-Aldrich, St. Louis, United States). SM22*α* expression was calculated by the corrected total cell fluorescence (CTCF) method as previously described [48] and plotted as the fold change relative to the flat PDMS control.

#### 2.3.2. Gene Expression Analysis

Total RNA was isolated using TRIzol reagent (Invitrogen Corp., Carlsbad, United States) according to the manufacturer's protocol. cDNA synthesis was performed using RevertAid™ First Strand cDNA Synthesis Kit (Thermo Scientific, Hemel Hempstead, United Kingdom), according to the manufacturer's protocol. The cDNA equivalent of 5 ng total RNA was used for amplification in 384-well microtitre plates in an ABI7900HT cycler (Applied Biosystems, Foster City, United States) using SYBR Green chemistry (Bio-Rad, Hercules, United States) with established primers (see Supplementary Table S1) to investigate myogenic genes *ACTA* and *TAGLN*. Cycle threshold (CT) values for individual reactions were determined using ABI Prism SDS 2.2 data processing software (Applied Biosystems, Foster City, United States) and normalized against *B2M* and *GAPDH* expression. All cDNA samples were amplified in duplicate. Relative expression was calculated using the ΔCt method. Data are presented as fold change relative to nonstimulated flat PDMS controls, obtained using the ΔΔCt method.

#### 2.3.3. Immunoblotting Analysis

Whole cell lysates from a 16 cm^2^ cell culture area were prepared in RIPA buffer (Thermo Scientific, Hemel Hempstead, United Kingdom) supplemented with 1% protease inhibitor cocktail (Sigma-Aldrich, St. Louis, United States). Lysed cells were collected in 1.5 ml microcentrifuge tubes and homogenized by sonication at 30 W for 10 s and centrifuged at 7500 g at 4°C for 5 min. The supernatant was collected, and protein concentration was determined using Bio-Rad DC protein assay (Bio-Rad, Hercules, United States), according to manufacturer's protocol. 20 *μ*g of protein was loaded on a 15% denaturing SDS polyacrylamide gel, separated by gel electrophoresis, and blotted onto nitrocellulose membrane (Bio-Rad, Hercules, United States) according to standard protocols. Blots were blocked in Odyssey Blocking Buffer (LI-COR Biosciences, Lincoln, United States) at 4°C overnight and incubated at room temperature for 2 h with primary antibodies against *α*SMA (1 : 1000; Abcam, Cambridge, United Kingdom), SM22*α* (1 : 1000; Abcam, Cambridge, United Kingdom), and GAPDH (1 : 1000; Abcam, Cambridge, United Kingdom) in Odyssey Blocking Buffer, supplemented with 0.1% Tween 20. Blots were then incubated with secondary antibodies for 1 h in Odyssey Blocking Buffer. Protein was detected using the Odyssey Infrared Imaging System (LI-COR Biosciences, Lincoln, United States). Densitometric analysis was performed using ImageJ software. Expression of target proteins was normalized to loading control, GAPDH. Data of each experimental condition are presented as fold change relative to the not TGF-*β*1-induced flat control.

### 2.4. Graphs and Statistical Analysis

All experimental data were obtained from three to five independent experiments with duplicate or triplicate. All data are presented as mean ± standard error of the mean (SEM). Graphs were plotted with GraphPad Prism (version 6.01; GraphPad Software Inc., La Jolla, United States) or Plotly (online version; Plotly Technologies Inc., Montreal, Canada). Statistical analyses were performed with GraphPad Prism, and interpolation data were calculated and plotted with Plotly as previously described [49]. For all experimental groups, two-tailed ratio paired *t*-test was performed for comparison against the nonstimulated flat PDMS control. For correlation purposes, the Pearson product-moment correlation was used.

## 3. Results

### 3.1. Surface Topography Characterization

**Figure 2:**
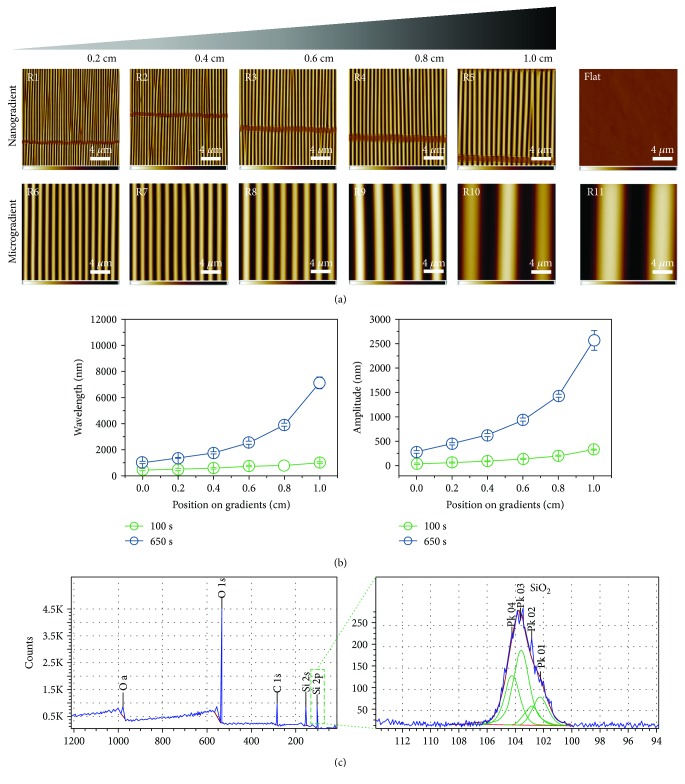
Surface topography characterization. (a) AFM images of the ten different ranges (small to large: R1-R11) of the two topographical directional gradients (nanometer scale and micrometer scale) along the PDMS substrate. Also shown is the flat PDMS control. Scale bars are 4 *μ*m and apply to all images. (b) Dependence of the wavelength and amplitude of created wrinkle gradients. The microgradient surface (blue) starts where the nanogradient surface (green) ends with respect to wavelength and amplitude. Data are reported as the mean ± standard deviation (*n* = 30). (c) XPS spectra of PDMS wrinkle gradients.

The surface of the PDMS directional topography gradients was analyzed using atomic force microscopy (AFM), with measurements acquired at 0.2 cm intervals of the 1 cm samples, with each sample being divided into five ranges (ranges R1 to R5 in the nanotopography gradient and ranges R6 to R11 in the microtopography gradient) (Figure 2). The flat PDMS control was also analyzed.

The topography sizes (wavelength and amplitude) increased from the side with the least exposure to the oxidative environment (air plasma) towards the side with the greatest exposure (at mask opening), as described in Table 1. In the nanogradient, features varied from 464 nm to 1012 nm in wavelength and 46 nm to 333 nm in amplitude, while the microgradient showed wavelengths from 1019 nm to 10990 nm and amplitudes of 286 nm to 3425 nm. Both amplitude and wavelength displayed a continuous gradual change. The flat control did not have any measurable features.

With regard to the chemical composition of the PDMS surfaces, Figure 2(c) shows the XPS spectra and confirmed overoxidized PDMS (SiO_2_) chemistry. The Si peaks at binding energies of 103.6 eV are consistent with Si^4+^, confirming the presence of SiO_2_ on the PDMS surface.

### 3.2. ASC Adherence, Alignment, and Differentiation Are Directed by Topographical Cues

**Figure 3:**
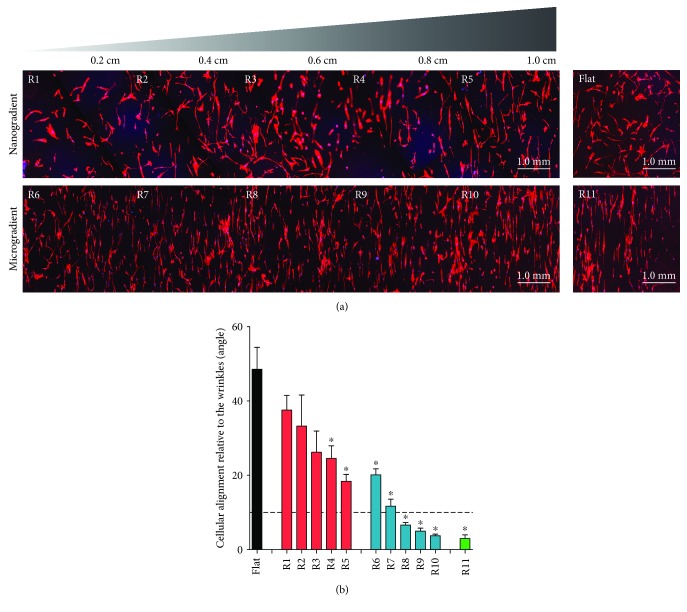
Cell alignment (24 h of culture). (a) Overview of the Phalloidin staining (red) for F-actin on the two different wrinkle gradients (R1-R5 and R6-R11), as well as on the flat PDMS control. (b) Cell alignment quantification as the mean cell angle relative to the directional topography. The dotted line represents the 10° cutoff defining the limit for cellular alignment. Black represents the flat PDMS control, red represents the nanotopography gradient, blue represents the microtopography gradient, and green represents R11; ^∗^ *p* < 0.05 vs. PDMS flat control. Values represent mean ± SEM of 3 independent experiments.

When analyzing cell alignment, all ranges beyond R3, i.e., all those larger than 731 nm in wavelength and 146 nm in amplitude, had statistically smaller angles in comparison to the flat control upon which the cells were randomly distributed (Figure 3). However, the only ranges upon which the cells could be considered aligned, according to the 10° limit, were ranges R8 to R11, thus all those larger than 1758 nm in wavelength and 630 nm in amplitude. These findings showed that the larger the features, the more efficient the cell alignment.

**Figure 4:**
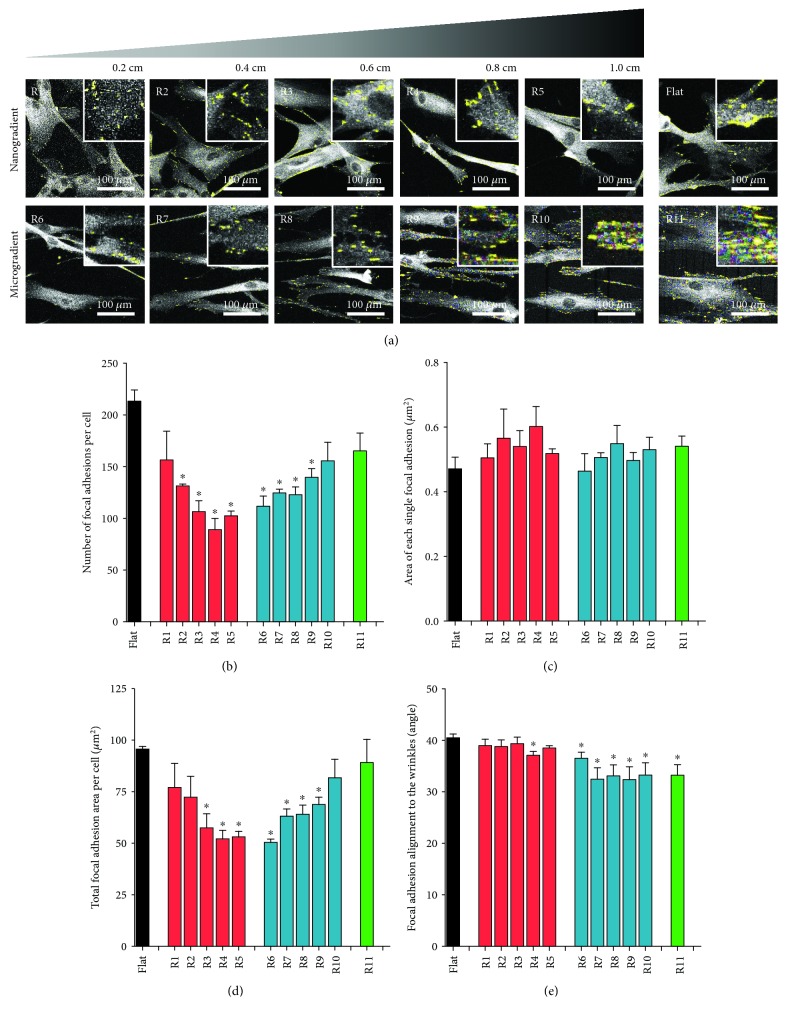
Focal adhesions (24 h of culture). (a) Fluorescence staining for vinculin (white) on the different wrinkle sizes of nano- (R1-R5) and microtopography (R6-R11) gradients, as well as on the flat PDMS control. Yellow dots represent the areas recognized as focal adhesions by the Focal Adhesion Analyze Server. (b) Number of focal adhesions per cell. (c) Area of each single focal adhesion. (d) Total focal adhesion area per cell. (e) Focal adhesion alignment to the directional topography. For all the graphs, black represents the flat PDMS control, red represents the nanotopography gradient, blue represents the microtopography gradient, and green represents R11; ^∗^ *p* < 0.05 vs. PDMS flat control. Values represent mean ± SEM of 3 independent experiments.

The characterization of focal adhesions is illustrated in Figure 4 (focal adhesions are represented by the yellow dots in Figure 4(a)). The number of focal adhesions per cell was highest on flat surfaces or the highest topography wavelengths (R10-R11, Figure 4). Interestingly, cells on the smallest topography (R1) had a similar number of focal adhesions to cells on flat PDMS. Towards the middle of the gradient, i.e., largest nanotopographies (R5) and smallest microtopographies R6), the number of focal adhesions per cell decreased by almost 50% (Figure 4(b)) with the lowest number of focal adhesions being observed between the 731 nm-784 nm wavelengths. Similarly, the total area of focal adhesions per cell decreased towards the middle of the gradient (Figure 4(d)). This was due only to the number of focal adhesions per cell because the average area of individual focal adhesions did not vary across the gradient (Figure 4(c)). The focal adhesions had aligned to the topography of the gradient in particular in the micrometer range (Figure 4(e), R6–R11).

**Figure 5:**
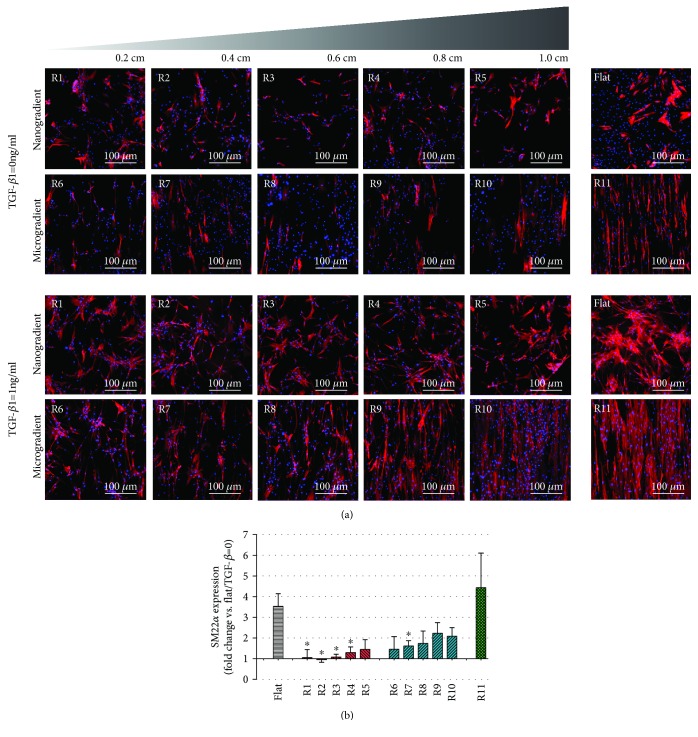
Cell differentiation (7 days of culture). (a) Fluorescence staining for SM22*α* (red) and DAPI (blue) in the different wrinkle sizes of nano- (R1-R5) and microtopography (R6-R11) gradients, as well as in the flat PDMS control. (b) SM22*α* expression in ASC induced by TGF-*β*1 as the fold change of the unstimulated flat PDMS control. Black represents the flat PDMS control, red represents the nanotopography gradient, blue represents the microtopography gradient, and green represents R11; ^∗^ *p* < 0.05 vs. TGF-*β*1-induced flat PDMS control. Values represent mean ± SEM of 3 independent experiments.

**Figure 6:**
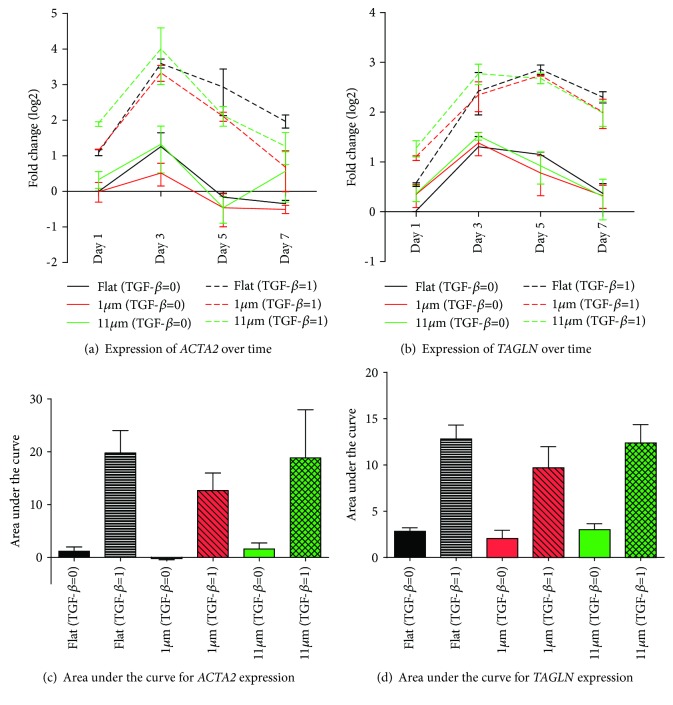
Influence of micrometer-sized topography and TGF-*β*1 stimulation on mesenchymal gene expression in adhered ASC over seven days. Expression of *ACTA2* (a) and *TAGLN* (b) increased over 3 days, independent of TGF-*β*1 stimulation and topography (flat vs. 1 or 11 *μ*m wavelength). For the net influence of TGF-*β*1 and topography, the area under the curve was determined for total expression of *ACTA1* (c) and *TAGLN* (d), respectively. This showed no differences between flat material and topographies, irrespective of TGF-*β*1. Values represent mean ± SEM of 3 independent experiments. Statistical analysis was performed comparing the flat topography to both of the linear topography patterns for both TGF-*β*1-stimulated and not stimulated groups.

**Figure 7:**
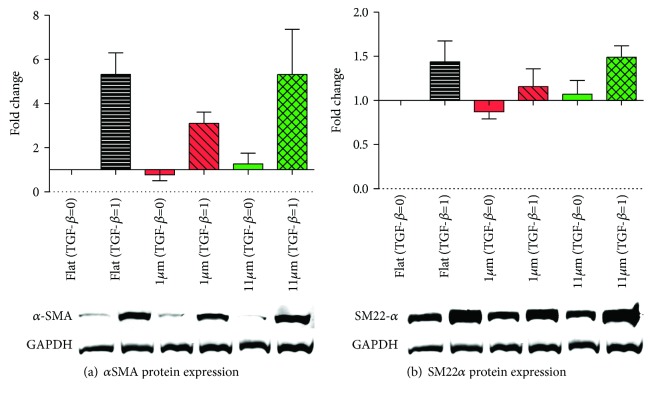
Protein expression of myogenic markers (7 days of culture). (a) Relative protein expression of *α*SMA with and without TGF-*β*1 induction in flat and uniform wrinkle (1 *μ*m and 11 *μ*m) topographies. Representative WB results are shown below the graph. (b) Relative protein expression of SM22*α* with and without TGF-*β*1 induction in flat and uniform wrinkle (1 *μ*m and 11 *μ*m) topographies. Representative WB results are shown below the graph. For all the graphs, black represents the flat PDMS control, red represents the 1 *μ*m uniform features, and green represents the 11 *μ*m uniform features. Values represent mean ± SEM of 3 independent experiments. Statistical analysis was performed comparing the flat topography to both of the linear topography patterns for both TGF-*β*1-stimulated and not stimulated groups.

TGF-*β*1-induced differentiation of ASC was suppressed on all topography gradients compared to controls, except for range R11 (Figure 5). Statistical differences in comparison to the flat control was only observed for topographies in ranges R1 to R4 and R7. For a more in-depth analysis, uniform topography features of 1 *μ*m and 11 *μ*m were used as representative substrates for ranges R5-R6 (the transition region between the nano- and the microgradient) and the largest features obtained (R11). These were compared to flat substrates to investigate ASC myogenic differentiation, analyzed both at the gene and the protein expression levels, respectively, by RT-qPCR and Western blot. A later stage myogenic marker, *α*SMA, was used together with SM22*α* for these evaluations. Gene expression peaked on the third day of culture for both *ACTA2* and *TAGLN* marker expression of which were reduced over time during the experiment (Figures 6(a) and 6(b)). The relative expression of the myogenic markers in the groups stimulated with TGF-*β*1 in comparison to the flat control showed that the change in *ACTA2* expression was more pronounced than *TAGLN* expression. This was not an unexpected finding due to the strong basal expression of TAGLN in ASC. Stimulation with TGF-*β*1 promoted differentiation of ASC, but also, spontaneous differentiation, in the absence of TGF-*β*, occurred, albeit at a lower level (Figure 6). It should be noted that this spontaneous differentiation waned at later time points as observed following normalization of the expression of both *ACTA2* and *TAGLN*. The analysis of the area under the curve delimited by the four time points analyzed (1, 3, 5, and 7 days) showed no difference among the myogenic marker expression, *ACTA2* and *TAGLN*, when comparing the two uniform aligned patterns and the flat PDMS control (Figures 6(c) and 6(d)). Protein expression, in turn, showed a distribution compatible with that predicted by the area under the curve for the gene expression (Figure 7), and no statistically significant difference was found.

### 3.3. Cell Alignment Is Accompanied by Focal Adhesion Alignment and Can Reduce the Minimum Expression of Focal Adhesions Required for ASC Myogenic Differentiation

**Figure 8:**
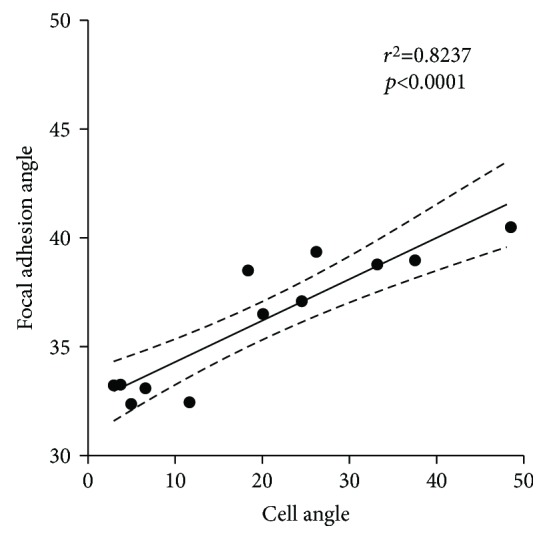
Scatter plot showing the correlation between cell and focal adhesion alignment. Pearson correlation, *r* ^2^ = 0.8237, *p* < 0.0001. Values represent the mean of 3 independent experiments. Data from the experiment using all 11 topographical ranges in which cells were cultured for 24 hours without TGF-*β*1.

**Figure 9:**
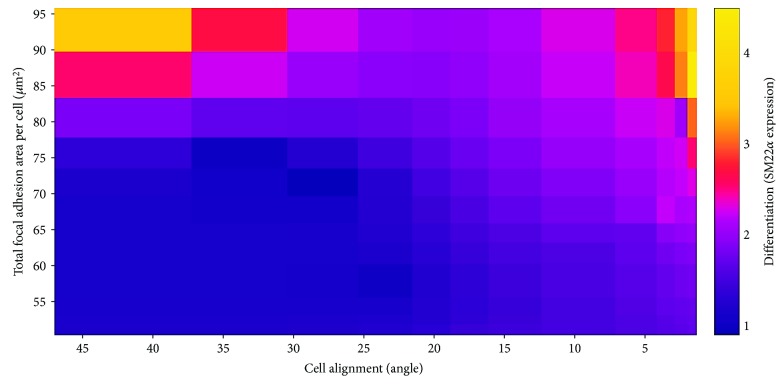
Heat map interpolating focal adhesion area per cell, cell alignment, and expression of SM22*α* data. Data for focal adhesions and cell alignment were from the experiment using all 11 topographical ranges in which cells were cultured for 24 hours without TGF-*β*1. Data for differentiation were from the experiment in which cells were cultured for 7 days under stimulation with TGF-*β*1, also with all 11 topographical ranges.

Taking into consideration the data from the experiment using all 11 topographical ranges in which cells were cultured for 24 hours without TGF-*β*1, the Pearson product-moment correlation coefficient was computed to assess the relationship between cell and focal adhesion alignment. There was a positive correlation between these two variables (*r* ^2^ = 0.8237, *p* < 0.0001) (Figure 8). By combining these data with the data from the differentiation experiment, in which cells were cultured for 7 days in the presence of TGF-*β*1, also with all 11 topographical ranges, a heat map in which the three readout parameters were integrated, i.e., interpolating total focal adhesion area per cell, cell alignment, and expression of SM22*α* (differentiation status), was generated. This output showed that these parameters interact with each other (Figure 9). Two regions of the heat map show a peak expression of SM22*α*. The first region (upper left) is the one presenting higher values of total focal adhesion area per cell, but with few or no alignments. The second (upper right) is the one characterized by greater alignment and higher values of focal adhesion area per cell. The second region displays lower values for focal adhesion area per cell as compared to the first region. In other words, the expression of focal adhesions correlates with myogenic differentiation of ASC, suggesting that a critical density of focal adhesions is required for ASC differentiation. The threshold of required focal adhesions, however, is reduced when cells are aligned.

## 4. Discussion

The current study shows that surface topography directs three pivotal processes that are required for ASC-driven vascular tissue engineering, i.e., cell adhesion, alignment, and differentiation. To the best of our knowledge, this is the first time that differentiation of vascular precursor cells is investigated in a single continuous topography gradient. We show that smaller-sized topographies inhibit TGF-*β*-induced myogenic differentiation of ASC. On the other hand, larger-sized topographies supported alignment and differentiation of ASC which is a prerequisite for topography-guided generation of ASC-derived SMC for blood vessel tissue engineering. Our results partly corroborate published literature, particularly regarding the successful differentiation in microscale topographies [38, 41]. In contrast to previous studies, however, this was also the first time that inhibition of ASC differentiation in submicroscale topography is described. Our findings did not show differences in cellular responses in the regions between ranges R5 and R6—representing features from 784 nm to 1373 nm in wavelength and from 209 nm to 452 nm in amplitude—suggesting that the region around 1 *μ*m is a transition area before which the directional topographic feature size defines the fate of ASC with regard to their differentiation.

Current literature on the influence of surface topography on myogenic differentiation of MSC is limited. The available studies either use single-sized aligned nanofibers [39, 41] and wrinkles [40], complex topographies with variable groove/ridge ratios [37], or a combination of nano- and micropatterns together [38]. Two studies demonstrated increased differentiation of MSC to the myogenic lineage on directional topographies with 250 nm, 450 nm, and 900 nm spacings [37, 40], while two other groups demonstrated increased differentiation on topographies with 6 *μ*m, 10 *μ*m, and 20 *μ*m features [38, 41]. The differentiation of MSC into other lineages, such as adipogenic and osteogenic, has been studied to a greater extent [35–37, 50]. In general, adipogenic differentiation is favored by larger features of a directional topography [35–37]; osteogenic differentiation is more efficient on smaller features [35, 36]. However, whether there is also a link between ASC focal adhesion expression and fat or bone differentiation, as exists for myogenic differentiation, remains to be investigated.

The effects of different topographies on the differentiation of MSC are explained, to some extent, by the influences of mechanotransduction, which are the processes through which cells sense mechanical stimuli and respond by converting them into biochemical signals via cell contact guidance [51]. Focal adhesions are one of the main players in mechanotransduction and, thus, are important components for defining stem cell fate [52]. It was previously shown that topography modulates mechanotransduction of stem cells and induces differentiation through focal adhesion kinase [40]. In addition, the geometrical maturation of focal adhesions is essential for mechanotransduction [53]. At the same time, it was shown that focal adhesion expression is reduced in submicroscale topographies [40, 54] and that focal adhesion maturation occurs at the microscale, but not at the submicroscale [53]. These reports are in accordance with the findings of the present study, which showed a reduced expression of focal adhesions followed by reduced differentiation of ASC to SMC. Our results demonstrate that the transition region between the nanometer and the micrometer regimes harbors particular features that prevent the formation of focal adhesions. Comparable results were also described for fibroblast to myofibroblast differentiation, which correlates with a reduced focal adhesion area and reduced differentiation subsequently [55]. In addition, our results show that smaller micropatterns reduced myogenic marker expression compared to bigger micropatterns [56]. Although we showed that mechanosensing differs according to the surface topography, changing cell plasticity and identification of the signaling pathways involved in this process were outside the scope of this study.

The direct relationship between focal adhesions and differentiation in an alignment scenario, as in the present study, is not possible to be inferred, because there is a combined effect of the presence of focal adhesions and the cell alignment in the differentiation of the cells. Thus, the analysis of the effect of both factors must be performed simultaneously. In order to achieve this, the heat map interpolating total focal adhesion area, cell alignment, and SM22*α* expression data (Figure 9) was created. This enabled us to observe that although the expression of focal adhesions is essential for the ASC differentiation, the threshold of required focal adhesions appears to be reduced when cells—and consequently focal adhesions—are aligned to their topographical environment. Thus, these data suggest that alignment, in parallel with focal adhesion expression, is also a crucial factor and possibly even the main driver for defining a cell's fate, which is probably closely linked to the mechanotransduction-mediated mechanisms. The increasing expression of SM22*α* with increasing alignment indicates a strong correlation between these two events irrespective of the number of focal adhesions present. The direction of the arrangement of the focal adhesions displays a similar behavior as the cell directionality, albeit it in a less prominent fashion. These findings indicate that the number of focal adhesions is of less importance for cell alignment and that the amount of focal adhesions does not correlate with the signal transduction from the cell surroundings to intracellular mechanisms. Most likely, the lower amount of focal adhesions is due to a matching in physical size of the topography and focal adhesions. Features much smaller than the focal adhesions will be perceived as planar as the focal adhesions will span many features simultaneously while features much larger allow focal adhesions to be formed between the features.

## 5. Conclusion

We show that micrometer-sized topographies are promising tools for driving adhesion, alignment, and TGF-*β*1-induced generation of vascular smooth muscle cells from ASC. Our unique topographical gradients also showed that nanometer-sized topographies inhibit ASC differentiation while adhesion was not affected.

## Figures and Tables

**Table 1:** Topography gradient ranges.

Topography	Range	Position on gradient (cm)	Wavelength (nm)	Amplitude (nm)
Nanotopography gradient	R1	0.0-0.2	464-483	49-62
	R2	0.2-0.4	483-562	62-96
	R3	0.4-0.6	562-731	96-146
	R4	0.6-0.8	731-784	146-209
	R5	0.8-1.0	784-1012	209-333

Microtopography gradient	R6	0.0-0.2	1019-1373	286-452
	R7	0.2-0.4	1373-1758	452-630
	R8	0.4-0.6	1758-2529	630-939
	R9	0.6-0.8	2529-3919	939-1430
	R10	0.8-1.0	3919-7121	1430-2561
	R11	>1.0	10990	3425

## Data Availability

The datasets generated during and/or analyzed during the current study are available from the corresponding author on reasonable request.

## Supplementary Materials

Supplementary Materials Supplementary Table S1: primer sequences of genes.
