# Supplementary material for: Directional Topography Influences Adipose Mesenchymal Stromal Cell Plasticity: Prospects for Tissue Engineering and Fibrosis
Source: Stem Cells Int. 2019 May 5;2019:5387850. doi: 10.1155/2019/5387850 (PMC6525798; doi:10.1155/2019/5387850)
Supplement: Supplementary Materials — Supplementary Table S1: primer sequences of genes. [file 5387850.f1.pdf]

SUPPLEMENTARY MATERIAL

Supplementary Table S1. Primer sequences of genes

**Gene**	**Primer Sequence (5' – 3')**	
B2M	F	TGCTGTCTCCATGTTTGATGTATCT
	R	TCTCTGCTCCCCACCTCTAAGT
GAPDH	F	AGCCACATCGCTCAGACAC
	R	GCCCAATACGACCAAATCC
ACTA2	F	CTGTTCCAGCCATCCTTCAT
	R	TCATGATGCTGTTGTAGGTGGT
TAGLN	F	CAAAGCCATCAGGGTCCTC
	R	TTCCAGACTGTTGACCTCTTTG
